# Loss of a Mycobacterial Gene Encoding a Reductase Leads to an Altered Cell Wall Containing β-oxo- Mycolic Acid Analogs and Accumulation of Ketones

**DOI:** 10.1016/j.chembiol.2008.07.007

**Published:** 2008-09-22

**Authors:** Apoorva Bhatt, Alistair K. Brown, Albel Singh, David E. Minnikin, Gurdyal S. Besra

**Affiliations:** 1 School of Biosciences, University of Birmingham, Edgbaston, Birmingham B15 2TT, UK

**Keywords:** CHEMBIO

## Abstract

Mycolic acids are essential components of the mycobacterial cell wall. In this study, we show that a gene encoding a reductase involved in the final step of mycolic acid biosynthesis can be deleted in *Mycobacterium smegmatis* without affecting cell viability. Deletion of *MSMEG4722* (ortholog of *Mycobacterium tuberculosis Rv2509*) altered culture characteristics and antibiotic sensitivity. The Δ*MSMEG4722* strain synthesized α-alkyl, β-oxo intermediates of mycolic acids, which were found esterified to cell wall arabinogalactan. While the precursors could not be isolated directly due to their inherent instability during base treatment, their presence was established by prior reduction of the β-oxo group by sodium borohydride. Interestingly, the mutant also accumulated unsaturated ketones, similar to tuberculenone from *M. tuberculosis*, which were shunt products derived from spontaneous decarboxylation of α-alkyl, β-oxo fatty acid precursors of mycolic acids.

## Introduction

**Figure 1:**
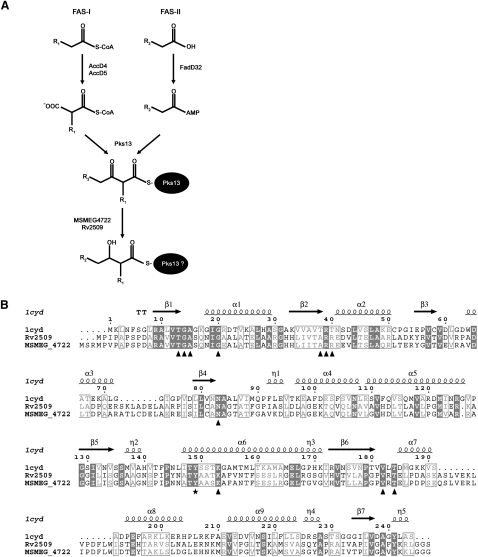
Mycolic Acid Reductases in Mycobacteria (A) Schematic representation of the post-FAS-II steps in mycobacterial mycolic acid biosynthesis. AccD4 and AccD5 are acyl-CoA carboxylases, while FadD32 is an acyl-AMP ligase. It is yet unclear whether the reduction of the β-oxo group occurs while the mycolic acid precursor in still attached to Pks13, or after release from Pks13 by a thioesterase. (B) Alignment of amino acid sequences of Rv2509 and MSMEG4722 with 1cyd (PDB file for the structure of *Mus musculus* carbonyl reductase with NADP and 2-propanol). α Helices and β sheets are indicated above the residues as coils and arrows, respectively. Residues essential for NAD/NADP binding are indicated by triangles, while the active-site residue is indicated with a star.

Mycolic acids are a major and essential component of the lipid-rich cell envelope of the human pathogen *Mycobacterium tuberculosis* and other related mycobacteria. Found either covalently attached to the terminal arabinose residues of the mycolyl arabinogalactan-peptidoglycan (mAGP) complex, or as the free glycolipids, trehalose monomycolate (TMM), trehalose dimycolate (TDM), and glucose monomycolate (GMM), these α-alkyl, β-hydroxyl long-chain fatty acids play an important role in reduced cell wall permeability (Brennan and Nikaido, 1995; Daffe and Draper, 1998; Gao et al., 2003; Jackson et al., 1999) and virulence (Bhatt et al., 2007; Dubnau et al., 2000; Gao et al., 2003; Glickman et al., 2000; Rao et al., 2006) of mycobacteria. In *M. tuberculosis*, a multifunctional type I fatty acid synthase (FAS-I) synthesizes C_16–18_ and C_24–26_ fatty acids in a bimodal fashion. The former is then channeled to a type-II, multienzyme complex called FAS-II, which, through its iterative reductive cycles, extends the acyl chain to long-chain meromycolic acids (C_56–64_) (Bloch, 1975, 1977; Brindley et al., 1969; Peterson and Bloch, 1977). Finally, a polyketide synthase, Pks13, catalyzes the Claisen condensation of a C_26_ fatty acid and a mero-acid to yield an α-alkyl, β-oxo acyl intermediate, which in turn is reduced to form a mature mycolic acid (Gande et al., 2004; Portevin et al., 2004) (Figure 1A). While earlier studies were focused on identifying genes encoding “core” FAS-II enzymes, little was known about the final, post-Pks13 step of mycolic acid biosynthesis: the reduction of the β-oxo group to a hydroxyl group leading to the formation of the mycolic acid motif. Unlike in mycobacteria, genes encoding enzymes involved in the biosynthesis of mycolic acids are nonessential in corynebacteria, facilitating the generation of null mutants. Recently, Lea-Smith et al. (2007) generated a mutant of *Corynebacterium glutamicum NCgl2385* that had a slow-growth phenotype, and produced corynomycolate precursors with a β-oxo group. In the same study, the authors also used bioinformatics to identify Rv2509, the *M. tuberculosis* ortholog of NCgl2385, as a possible candidate for reduction of the mycolic acid motif (Lea-Smith et al., 2007). *Mycobacterium smegmatis* has often been used as a surrogate for *M. tuberculosis* when studying biosynthetic pathways. The fast-growing, nonpathogenic *M. smegmatis* strain is particularly useful in studying cell wall biosynthesis genes, since it can tolerate deletion of some genes that are essential in *M. tuberculosis* (Amin et al., 2008; Escuyer et al., 2001). Moreover, while the two species differ in mero-chain modifications, core enzymes involved in mycolate biosynthesis are interchangeable (Brown et al., 2007; Parish et al., 2007). We thus chose to address the role of *Rv2509* in mycobacterial mycolic acid biosynthesis by generating a deletion mutant of *MSMEG4722*, the *M. smegmatis* homolog of *Rv2509*.

## Results

### *MSMEG4722* and *Rv2509* Encode Proteins Structurally Similar to Short-Chain Reductases/Dehydrogenases

Through the use of bioinformatics, Lea-Smith et al. (2007) identified *M. tuberculosis* Rv2509 as the homolog of NCgl2385, the *C. glutamicum* reductase involved in mycolic acid motif formation. The closest match for Rv2509 in the *M. smegmatis* mc^2^155 genome was the putative protein MSMEG4722 (Figure 1B). Both predicted proteins contained conserved active site residues and residues for NAD/NADP binding (Figure 1B). Predictions of the three-dimensional (3D) structures of proteins often give insights into potential catalytic properties. We used the @TOME server to screen for known structures of proteins that were predicted to be most closely related to Rv2509 (Douguet and Labesse, 2001). Predictions of E values from TITO and 3D-PSSM servers (−121140 and 1.23e-02, respectively) strongly suggested that 1cyd (*Mus musculus* carbonyl reductase complexed with NADPH and 2-propanol) was the closest match to Rv2709 (22% sequence identity). Through use of the 1cyd coordinates and the FUGUE server (Shi et al., 2001), we generated an in silico 3D structure of Rv2509. The predictions revealed similar 3D structural folds for 1cyd, Rv2509, and the *Escherichia coli* fatty acid reductase, FabG (data not shown). Additionally, when the NADPH moiety from 1cyd was superimposed in the predicted NADP-binding fold of Rv2509, the predicted distances between the conserved residues and the cofactor showed a fit similar to that seen in 1cyd (data not shown). These data suggest that Rv2509 was likely an NAD/NADP-dependent mycobacterial reductase. As outlined above, the homologous *M. smegmatis* gene *MSMEG4722* was chosen for further analysis.

### Deletion of *MSMEG4722* in *M. smegmatis* mc^2^155 Alters Culture Characteristics and Sensitivity to Antibiotics

**Figure 2:**
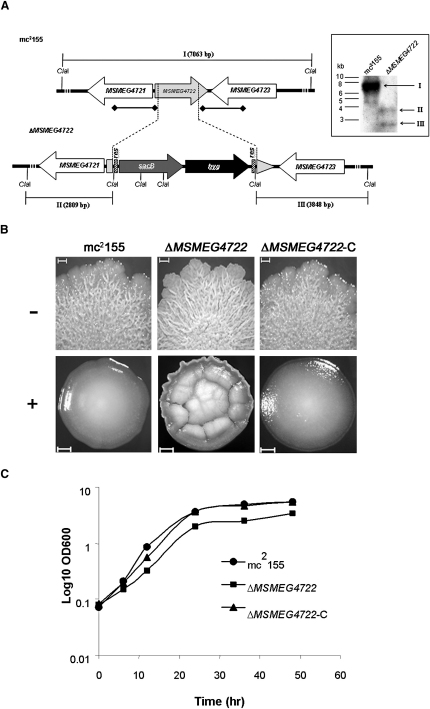
Generation of a *MSMEG4722* Null Mutant (A) Map of the *MSMEG4722* region in the parental *M. smegmatis* strain mc^2^155 and its corresponding region in the Δ*MSMEG4722* mutant. *res*, γδ resolvase site; *hyg*, hygromycin resistance gene from *Streptomyces hygroscopicus*; *sacB*, sucrose counterselectable gene from *Bacillus subtilis*. Digoxigenin-labeled probes were derived from ∼1 kb upstream and downstream flanking sequences that were used to construct the knockout plasmid, and are indicated by thick lines with diamond-shaped ends. ClaI-digested bands expected in a Southern blot are indicated in roman numerals with sizes in parentheses. The inset shows the Southern blot of ClaI-digested genomic DNA from the two strains with expected bands indicated by arrows. (B) Colonies of wild-type (mc^2^155), mutant (Δ*MSMEG4722*), and complemented (Δ*MSMEG4722*-C) strains on TSB-agar (−) or TSB-agar + 0.05% Tween-80 (+). Colony growth shown in upper row was obtained by inoculating 5 μl of a broth culture on the agar plate, while the lower row shows pictures of a single isolated colony of each strain. Scale bar = 1 mm. (C) Growth curve of wild-type (mc^2^155), mutant (Δ*MSMEG4722*), and complemented (Δ*MSMEG4722*-C) strains in TSB.

In order to study the role of *MSMEG4722* in mycolic acid motif formation, we deleted *MSMEG4722* in *M. smegmatis* mc^2^155 by specialized transduction (Bardarov et al., 2002) (Figure 2A). The ability to generate a null mutant indicated that *MSMEG4722* was not essential for the viability of *M. smegmatis* mc^2^155. Loss of *MSMEG4722* had a remarkable effect on the colony morphology of *M. smegmatis* mc^2^155 on tryptic soy broth (TSB) agar. While the colonies of the parental, wild-type strain mc^2^155 were glossy, those of the mutant strain Δ*MSMEG4722* appeared to have a dry surface (Figure 2B). The change was more apparent when the strains were grown on TSB agar supplemented with Tween-80. Unlike colonies of the parental strain, mc^2^155, which had a smooth surface, colonies of Δ*MSMEG4722* had an irregular, convoluted surface (Figure 2B). The Δ*MSMEG4722* mutant also showed a slightly slower growth rate than the parental mc^2^155 strain (Figure 2C; the OD_600_ values at 24h correspond to 2 × 10^8^ and 10^7^ colony forming units/ml for mc^2^155 and Δ*MSMEG4722*, respectively). This was not surprising, as a similar growth defect was observed when the homologous gene was deleted in *C. glutamicum*, and a genome-wide transposon screen predicted that the loss of the homologous gene in *M. tuberculosis* would result in a slow-growth phenotype (Lea-Smith et al., 2007; Sassetti et al., 2003). Additionally, when grown in Luria-Bertani broth (LB), the mutant showed an increased sensitivity to the lipophilic antibiotic rifampicin (minimum inhibitory concentrations [MIC] = 0.125 μg/ml) as compared with the parental strain mc^2^155 (MIC = 16 μg/ml), but not to hydrophilic antibiotics, such as isoniazid and ethambutol. Wild-type characteristics were restored on complementation of the Δ*MSMEG4722* mutant with plasmid-borne *MSMEG4722*, indicating that the observed phenotypes in the mutant strain were solely due to the loss of *MSMEG4722* (Figures 2B and 2C).

### The Δ*MSMEG4722* Mutant Failed to Synthesize Mature Mycolic Acids

**Figure 3:**
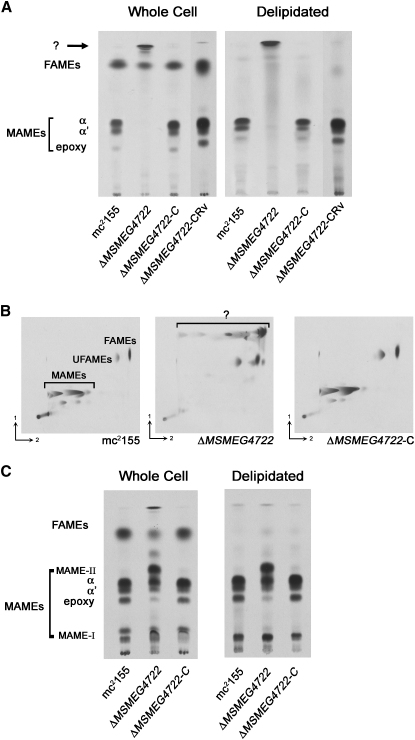
TLC Analysis of Mycolic Acid Methyl Esters (A) TLC analysis of MAMEs extracted from mc^2^155, Δ*MSMEG4722*, Δ*MSMEG4722*-C, and Δ*MSMEG4722*-CRv strains. The rapidly migrating species observed in Δ*MSMEG4722* are indicated by a question mark. FAMEs, fatty acid methyl esters. (B) 2D Ag^+^-TLC of MAMEs extracted from mc^2^155, Δ*MSMEG4722*, and Δ*MSMEG4722*-C. UFAMEs, unsaturated fatty acid methyl esters. (C) TLC analysis of MAMEs extracted from cells pretreated with NaBH_4_.

The predicted role of *MSMEG4722* in mycolic acid motif formation and the observed changes in the colony morphology of the Δ*MSMEG4722* mutant prompted us to examine mycolic acids in the mutant strain. If MSMEG4722 was indeed the reductase catalyzing the conversion of the post-Pks13, α-alkyl, β-oxo fatty acyl intermediate, then the Δ*MSMEG4722* mutant would be expected to accumulate this unreduced intermediate of mycolic acid biosynthesis (Figure 1A). A standard procedure for release of mycolic acids from mycobacteria involves base hydrolysis of cells with tetrabutyl ammonium hydroxide (TBAH). This is followed by phase-transfer catalyzed derivatization with methyl iodide that results in the formation of mycolic acid methyl esters (MAMEs) (Dobson et al., 1985), which are analyzed by thin-layer chromatography (TLC). Following base treatment and derivatization, [^14^C]-labeled extracts obtained from strains were analyzed by TLC. While α, α′ and epoxy MAMEs were present in the parental mc^2^155 strain, all three species were missing in the extract from the Δ*MSMEG4722* strain (Figure 3A). Instead, the mutant strain showed the accumulation of a product(s) with a higher retardation factor (*R_f_*) migrating above the fatty acid methyl esters (FAMEs). When extracts from the mutant strain were analyzed by 2D-Ag^+^-argentation TLC, the rapidly migrating species resolved into multiple subspecies in the second, Ag^+^-containing dimension, indicating the presence of multiple species differing in degrees of unsaturation (Figure 3B). An identical result was obtained for extracts from delipidated cells that only contain cell wall-bound mycolates (Figure 3A), indicating that the observed changes in mycolate profiles applied to both total and specifically cell wall-bound mycolates.

Mycolic acid biosynthesis was restored in the mutant strain following complementation with not only *MSMEG4722*, but also *Rv2509*, indicating that Rv2509 was a functional homolog of *MSMEG4722* in *M. tuberculosis* (strains Δ*MSMEG4722*-C and Δ*MSMEG4722*-CRv, respectively; Figure 3A).

### The Δ*MSMEG4722* Mutant Accumulates Precursors of Mycolic Acids in the Cell Wall

The complete absence of α, α′ and epoxy MAMEs and the appearance of rapidly migrating species in extracts of the Δ*MSMEG4722* mutant suggest that these rapidly migrating species may either be precursors of mycolates or decomposition products of precursors generated as a result of the extraction procedure. The latter seemed more likely, as base treatment of an unreduced α-alkyl, β-oxo fatty acid precursor, containing two oxo groups in close proximity, has been shown to result in the generation of a palmitone-like decomposition product in corynebacteria (Walker et al., 1973). Such decomposition products would be expected to migrate similarly to the rapidly migrating species that were observed in the extracts of the mutant strain. To confirm whether unreduced precursors of α, α′ and epoxy mycolic acids were present in the mutant strain, cells were pretreated with sodium borohydride (NaBH_4_). This pretreatment has little effect on normal mycolates, but results in the reduction of the β-oxo group in putative α-alkyl, β-oxo fatty acyl intermediates, resulting in the formation of α-alkyl, β-hydroxy products, viz. mature mycolic acids. Thus, if the α-alkyl, β-oxo fatty acid precursors of α, α′ and epoxy mycolic acids did exist in the mutant strain, pretreatment with NaBH_4_ would be expected to convert these precursors into α, α′ and epoxy mycolic acids, and TLC analysis of TBAH-treated, methylated extracts from NaBH_4_-pretreated Δ*MSMEG4722* cells would show the presence of α, α′ and epoxy MAMEs due to the prior reduction of the β-oxo group. When extracts of NaBH_4_-pretreated cells were analyzed by TLC, species migrating with the same *R_f_* values as α, α′ and epoxy MAMEs were observed (Figure 3C). However, additional MAMEs were also present in the extracts. Two closely migrating species had a very low *R_f_* value, and were detected in total mycolates strains from all strains (MAME-I; Figure 3C), while another with an *R_f_* value slightly greater than α-MAMEs was seen only in extracts from the mutant strain (MAME-II; Figure 3C).

MAME-I corresponded in chromatographic migration to two hydroxylated artifacts, characterized previously in acid methanolysates of mycobacteria having epoxy mycolates (Minnikin et al., 1982). Similar hydroxylated artifacts would be expected by NaBH_4_ reduction of the epoxy group in epoxy mycolates. Mass spectroscopic (MS) analysis confirmed these findings with molecular sizes detected in all strains corresponding to those of α, α′, epoxy MAMEs, and to the reduced, hydroxylated products of epoxy MAMEs (Table 1). Similar results were obtained when the analyses were performed on delipidated cells, indicating that the α-alkyl, β-oxo fatty acyl precursors of mycolates from *M. smegmatis* were esterified to the AG in the cell wall (Figure 3C). However, in this case, only a single MAME-I hydroxylated component was produced on NaBH_4_-reduction of the delipidated cells (Figure 3C).

The NaBH_4_-reduction of the β-oxo group, in a mycolate precursor, would result in two diastereoisomers of MAMEs, which would migrate differently on TLC (Minnikin and Polgar, 1966). MAME-II, therefore, was likely to be a mycolate β-epimer and, on matrix-assisted laser desorption ionization-time of flight/MS (MALDI-TOF/MS), the molecular size was found to be identical to that of α-MAMES. Indeed, purified MAME-II comigrated with a known standard for α-MAME β-epimer (data not shown). These results showed that the Δ*MSMEG4722* mutant, due to loss of mycolyl reductase function, failed to make mature mycolic acids, and instead synthesized the α-alkyl, β-oxo fatty acyl precursors of α, α′ and epoxy mycolates that were transported and subsequently esterified to the reducing termini of the AG complex.

### Lipid Analysis Revealed Accumulation of a Nonpolar Lipid in the Δ*MSMEG4722* Mutant

**Figure 4:**
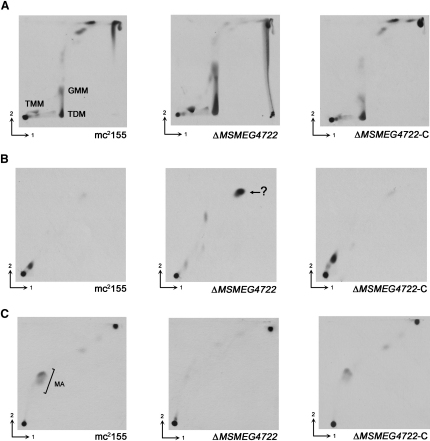
2D-TLC Analysis of Apolar Lipids Extracted from mc^2^155, Δ*MSMEG4722*, and Δ*MSMEG4722*-CStrains (A) Direction 1, chloroform:methanol:water, 100:14:0.8 (v/v); direction 2, chloroform:acetone:methanol:water, 50:60:2.5:3 (v/v). GMM, glucose monomycolate; TDM, trehalose dimycolate; TMM, trehalose monomycolate. (B) Direction 1, petroleum ether:acetone, 98:2 (v/v, thrice); direction 2, toluene:acetone, 98:2 (v/v). Lipid-Y is indicated by an arrow and question mark. (C) Direction 1, chloroform:methanol, 96:4 (v/v); direction 2, toluene:acetone, 80:20 (v/v). MA, mycolic acids.

**Figure 5:**
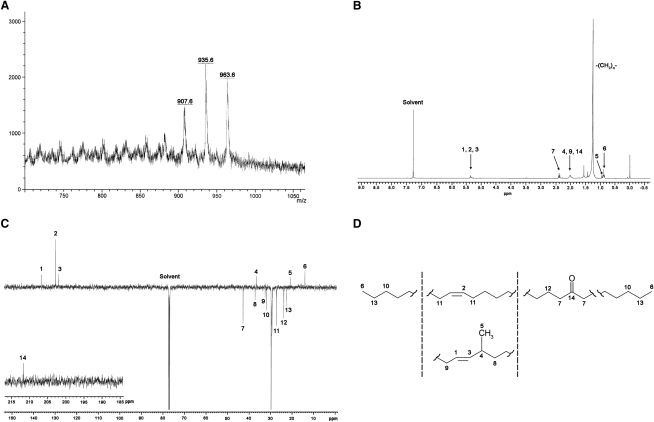
Structural Analysis of Lipid-Y (A–D) (A) MALDI-TOF/MS, (B) ^1^H-NMR, and (C) ^13^C-NMR of purified Lipid-Y. Characteristic shifts are labeled as numbers, and the structures they represent are indicated in (D).

Polar and nonpolar lipids labeled with [^14^C]-acetate were extracted from mc^2^155, Δ*MSMEG4722*, and Δ*MSMEG4722*-C strains and analyzed by 2D-TLC (Dobson et al., 1985). Interestingly, the lipids corresponding to TMM and TDM in mc^2^155 were replaced by two lipids with slightly altered mobilities in the mutant strain (Figure 4A). Both lipids had a slightly higher *R_f_* value (*R_f_* = 0.147 and *R_f_* = 0.39) than parental TMM and TDM (*R_f_* = 0.117 and *R_f_* = 0.36) in direction 1. This altered mobility was likely due to esterification of the α-alkyl, β-oxo mycolic acid precursors (instead of mature mycolic acids) to trehalose in the mutant strain, as was observed in the *C. glutamicum* mutant (Lea-Smith et al., 2007). Furthermore, the mutant strain showed accumulation of a nonpolar species accompanied by a total loss of free mycolic acids (Figures 4B and 4C). This lipid species, referred to as Lipid-Y, did not stain with Dittmer-Lester reagent or with α-napthol-sulfuric acid, indicating the absence of phosphate groups and carbohydrates (data not shown). Following purification by preparative TLC, Lipid-Y was characterized by MALDI-TOF/MS and nuclear magnetic resonance (NMR) (Figure 5). Three species of *m/z* 907.6, 935.6, and 963.6 were observed in the mass spectra (Figure 5A), indicating a difference of *m/z* 28 between each species. Further, ^1^H-NMR and ^13^C-NMR revealed a signal characteristic of −CH_2_− groups (1.3 and 30 ppm, respectively), and indicated the presence of alkyl chains differing (CH_2_)_2_ units. Additionally, ^1^H-NMR and ^13^C-NMR provided evidence of the presence of *cis* and/or *trans* double bonds (Figures 5B–5D; 1, 2, 3), with the latter possessing an adjacent methyl branch (Figures 5B–5D; 5) and a keto group (Figures 5C and 5D; 14), suggesting that Lipid-Y was a mixture of *cis*- and *trans*-unsaturated long-chain ketones (Figure 5D). Based on the masses obtained by MALDI-TOF/MS, these unsaturated, branched ketones were of chain lengths C_62_, C_64_, or C_66_. Since the accumulation of Lipid-Y was accompanied by a loss of mycolates (and no detectable free α-alkyl, β-oxo mycolate precursors), it seemed likely that the ketones that comprised Lipid-Y were derived from free β-oxo precursors. Free α-alkyl, β-oxo mycolate precursors could undergo decarboxylation to form ketones. If this was the case, then the oxo group would be situated between alkyl chains that originate from a meroacid on one side and the α branch on the other. Through the use of electron impact-MS (EI-MS), we were able to confirm the presence of a fragment of *m/z* 351, corresponding to the α chain (C_22_) plus a carbonyl group. In addition, the detection of fragments of *m/z* 546, 574, and 602, corresponding to C_39_, C_41_, and C_43_ monounsaturated alkyl chains, respectively, further substantiated our findings.

## Discussion

With the exception of KasB, all enzymes involved in the biosynthesis of mycolic acids in mycobacterial species are encoded by essential genes (Bhatt et al., 2005; Brown et al., 2007; Parish et al., 2007; Portevin et al., 2004; Sacco et al., 2007). However, we were able to generate a viable null mutant of *MSMEG4722*, the gene that encodes the reductase involved in mycolic acid motif synthesis in *M. smegmatis*. This was not entirely surprising, because global transposon mutagenesis screens predicted insertions in *Rv2509*, the *M. tuberculosis* homolog of *MSMEG4722*, to result in a slow-growth phenotype. Indeed, the Δ*MSMEG4722* mutant did exhibit a slow growth rate similar to that observed in *C. glutamicum* (Lea-Smith et al., 2007).

Alkaline hydrolysis of the parental strain *M. smegmatis* mc^2^155 released α−, α′− and epoxy mycolates, as expected, but hydrolyzates of the Δ*MSMEG4722* mutant showed no evidence of mycolates (Figure 3A). Instead, hydrolyzates of the mutant Δ*MSMEG4722* showed the presence of rapidly migrating components (Figures 3A and 3B, labeled “?”). If the Δ*MSMEG4722* mutant was accumulating α-alkyl, β-oxo mycolate precursors, alkaline hydrolysis would produce unstable β-oxo acids, which would lose carbon dioxide to yield long-chain ketones. Through an alternative approach, we confirmed the presence of the α-alkyl, β-oxo fatty acyl precursors by converting them to α-alkyl, β-hydroxy fatty acids (mycolates) by prior reduction of bound mycolates with NaBH_4_. This resulted in the appearance of α, α′ and epoxy-MAMEs in extracts of both whole and delipidated cells from the mutant strain (Figure 3C).

As expected (Minnikin and Polgar, 1966), reduction of the β-oxo mycolate precursors gave a mixture of separable diastereoisomers, and the presence of the β-epimer of α-MAME was clearly seen (MAME-II; Figure 3C). It would be expected that the β-epimers of α′− and epoxy mycolates would also be produced, but such minor compounds would not be readily seen on 1D-TLC (Figure 3C). The epoxy function is also susceptible to NaBH_4_ reduction and isomeric hydroxylated derivatives were identified (MAME-I; Figure 3C), corresponding to two artifacts previously characterized in acid methanolysates (Minnikin et al., 1982). However, reduction of the delipidated cells gave only the most polar hydroxylated derivative (Figure 3C). This strongly suggests that access of NaBH_4_ to cell wall-bound epoxy mycolates was restricted. It has recently been shown that keto mycolates from *Mycobacterium bovis* BCG adopt a folded “W” conformation (Villeneuve et al., 2007) with the keto group in a similar hydrophilic environment as the hydroxy acid unit. It is reasonable to suggest that bound epoxy mycolic acids might also prefer such a folded “W” conformation that could direct the access of NaBH_4_ in a regiospecific manner, resulting in the formation of only a single hydroxylated derivative. It is notable that the covalently bound β-oxo precursors in the Δ*MSMEG4722* mutant also produce only the more polar derivative. This would suggest that β-oxo precursors also fold in the same way as intact mycolates, indicating that a β-hydroxy group is not an absolutely essential prerequisite for folding in a “W” conformation. Indeed, the fact that Δ*MSMEG4722* mutant cells are viable, with β-oxo mycolate analogs in their envelopes, strongly suggests that an exchange of a native β-hydroxy group for an unnatural β-oxo unit is permitted. However, as discussed later, the Δ*MSMEG4722* mutant cells were more permeable to lipophilic antibiotics, and colony morphology was affected. Further studies will be needed to clarify these intriguing observations.

Additionally, replacement of TMM and TDM with derivatives with slightly altered TLC mobility in Δ*MSMEG4722* (Figure 4A) suggest that the α-alkyl, β-oxo mycolate precursors were also esterified to trehalose. Indeed, we obtained similar results for MAME analysis from extracts of whole cells (which contain wall-bound and trehalose-bound mycolates) as well as delipidated cells (which contain only wall-bound mycolates), demonstrating that mycolic acids in both the mAGP complex and in TMM/TDM were replaced by the α-alkyl, β-oxo fatty acid precursors.

While the loss of the reductase was expected to generate precursors of mycolic acids, it was surprising that the α-alkyl, β-oxo fatty acids were associated with the cell wall. These data suggest that mycobacterial components involved in the processing, transport, and subsequent transfer of mycolic acids to the cell wall (including the hypothetical mycolyl transferases I and II and the proteins of the antigen 85 complex [Takayama et al., 2005]) were probably able to do the same with the α-alkyl, β-oxo fatty acid intermediates. In contrast, Lea-Smith et al. (2007) reported reduced levels of AG-mycolylation in the *C. glutamicum* reductase mutant (Δ*NCgl2385*). Corynomycolate derivatives released from mutant cell wall were distinct from wild-type corynomycolates, and showed an ∼80% reduction in abundance. However, the extraction method used in this previous study involved acid-methanolyis. In light of our findings, it is likely that, rather than a reduction in mycolylation, the Δ*NCgl2385* mutant contained equally abundant α-alkyl, β-oxo corynomycolate precursors esterified to the AG, which were not detected by GC and MS because of decomposition resulting from acid-methanolysis.

Incorporation of the α-alkyl, β-oxo fatty acid precursors, instead of mycolic acids in the cell wall, had a significant impact on the characteristics of the cell wall of *M. smegmatis*, rendering the mutant strain more susceptible to lipophilic antibiotics due to an increased permeability. The presence on a β-oxo rather than a β-hydroxyl group also affected the colony morphology of the mutant strain, presumably due to changes in the hydrophobicity of the outer surface of the bacterial cells.

A key distinction between the reductase mutants of *C. glutamicum* and *M. smegmatis* was the accumulation of an unusual lipid, Lipid-Y, in the latter. MS and NMR analyses of purified Lipid-Y revealed it to be a mixture of unsaturated, branched ketones. Similar ketones were detected in strains of *M. tuberculosis* (tuberculenone) and *Corynebacterium diptheriae* (Asselineau, 1954; Pudles and Lederer, 1954). The total number of carbons (C_60_ ± C_3_) in the monounsaturated *M. tuberculosis* ketone, tuberculenone is similar to those of Lipid-Y (C_62_–C_66_) in the *MSMEG4722* mutant. However, tuberculenone was characterized before the advent of mass spectrometry (Asselineau, 1954), so precise comparisons are not meaningful. It has been suggested that ketones like tuberculenone are derived from decarboxylation of the α-alkyl, β-oxo fatty acid intermediates of mycolic acids (Asselineau, 1966). In addition to cell wall-bound and glycolipid-associated mycolates, mycobacteria also contain free mycolic acids, and would be expected to contain some transient, unreduced mycolic acid intermediates at any given time. It is likely that tuberculenone is derived from these intermediates. In the Δ*MSMEG4722* mutant, however, no free mycolic acids were detected (Figure 4C). Instead, an accumulation of α-alkyl, β-oxo fatty acid intermediates would be expected to occur. Free acids of such intermediates would then undergo decarboxylation to form the ketones that comprise Lipid-Y. Through the use of EI-MS, we were able to confirm that this was indeed the case.

Environmental mycobacteria are known to alter cell wall mycolate composition in response to growth substrates, resulting in a more hydrophobic wall when grown in the presence of hydrophobic substrates (Wick et al., 2002). It is not clear whether mycolate reduction is regulated by environmental factors, but our studies here have shown that loss of the β-hydroxy mycolate motif reduction alters cell wall hydrophobicity. Whether this has an effect in vivo (in the case of *M. tuberculosis*) remains to be studied.

In conclusion, our results clearly demonstrate that MSMEG4722 is the reductase involved in generation of the mycolic acid motif in *M. smegmatis*. The loss of this function is not lethal, allowing cell wall incorporation of β-oxo mycolate analogs, but affecting the growth characteristics of the bacterium.

## Significance

**Mycolic acid biosynthesis is essential for mycobacterial survival and many antituberculosis drugs, such as isoniazid, ethionamide, and thiolactomycin, target enzymes of this exclusive pathway (****Banerjee et al., 1994; Kremer et al., 2000****). Interestingly, MSMEG4722, which catalyzes the final step in mycolic acid biosynthesis in *Mycobacterium smegmatis*, is nonessential, and α-alkyl, β-oxo mycolate precursors are attached to arabinogalactan and trehalose. However, we have demonstrated that loss of function does cause major changes in the cell wall of *M. smegmatis*, making it more susceptible to lipophilic antibiotics, such as rifampicin. By extension, loss of *Rv2509*, the *Mycobacterium tuberculosis* homolog, would be expected to have a bearing not only on susceptibility to antibiotics, but also on virulence, as strains of *M. tuberculosis* with altered mycolic acids are highly attenuated (****Bhatt et al., 2007; Dubnau et al., 2000; Glickman et al., 2000****), highlighting the potential of Rv2509 as a “secondary” drug target. Our studies also shed some light on the post-fatty acid synthase-II/Pks13 processing, transport, and transfer of mycolic acids to their location in the cell envelope. The replacement of mycolic acids in the cell wall by the α-alkyl, β-oxo fatty acid precursors suggested that post-Pks13 reduction of the β-oxo group was not necessary for the subsequent processing pathways.**

## Experimental Procedures

### Bacterial Strains, Phages, Plasmids, and Culture Conditions

All plasmids, phages, and bacterial strains used in this study are shown in Table 2. Strains of *E. coli* were cultured in LB (Difco). *M. smegmatis* strains were grown in either LB broth or tryptic soy broth (TSB; Difco), each containing 0.05% Tween-80. TSB-agar was prepared by adding 1.5% agar to TSB prior to autoclaving. For *M. smegmatis*, hygromycin (100 μg ml^−1^) or kanamycin (20 μg ml^−1^) was used for selection, while hygromycin (150 μg ml^−1^) or kanamycin (40 μg ml^−1^) was used for selecting recombinant *E. coli* strains. Determination of MIC of antibiotics was done in LB with the Alamar blue assay (Franzblau et al., 1998).

### Bioinformatics

Sequence alignments were determined with BLAST or EBI ClustalW (Chenna et al., 2003), and rendered with the EScript 2.2 Web server. Structural predictions were performed with the @TOME server (Douguet and Labesse, 2001), and modeling performed with the FUGUE Web server (http://tardis.nibio.go.jp/fugue/align.php). PyMOL (DeLano Scientific) was used to create POV scenes followed by rendering by POV-Ray.

### Construction of a *MSMEG4722* Null Mutant

Approximately 1 kb sequences of the upstream and downstream regions of *MSMEG4722* were PCR-amplified from *M. smegmatis* mc^2^155 genomic DNA with the primer pairs MS4722LL (5′-TTTTTTTTCCATAAATTGGTGGCCAGCAGGT AGTAGACG-3′) and MS4722LR (5′-TTTTTTTTCCATTTCTTGGAGTTCGGTGGCCAACG CTTC-3′), and MS4722RL (5′-TTTTTTTTCCATAGATTGGTGGATCGACACCGAGTAC AC-3′) and MS4722RR (5′-TTTTTTTTCCATCTTTTGGAAACTGATCCGCTCCAAGGG-3′), respectively (all primers had Van91I recognition sites incorporated at the 5′ end). The PCR fragments were digested with Van91I and directly cloned into Van91I-digested p0004S (gift from T. Hsu and W.R. Jacobs Jr., Albert Einstein College of Medicine, NY). Recombinant plasmids obtained after transforming *E. coli* TOP-10 cells were digested with Van91I for confirmation and sequenced. One plasmid, pΔ*MSMEG4722*, was linearized by PacI digestion and packaged into the temperature-sensitive mycobacteriophage phAE159, as previously described (Bardarov et al., 2002), to yield phasmid DNA of the knockout phage phΔ*MSMEG4722*. Generation of high-titer phage particles and specialized transduction were performed as described previously (Bardarov et al., 2002). Allelic exchange in hygromycin-resistant transductants was confirmed by Southern blot.

### Construction of Complemented Strains

*MSMEG4722* was PCR amplified from *M. smegmatis* mc^2^155 genomic DNA with the primers MS4722-U (5′-GCAGGATCCAATGAGCCGCATGCCAGTACCCG-3′) and MS4722-D (5′-GCAGAATTCCTAACCGCCGCCGAGCTTCTTG-3′) and cloned into the *E. coli*-*Mycobacterium* shuttle plasmid pMV261 with the primer-incorporated BamHI and EcoRI sites, to yield the recombinant plasmid pMV261-*MSMEG4722*. In a similar fashion, the plasmid pMV261-*Rv2509* was constructed with *Rv2509*, which was PCR amplified from *M. tuberculosis* H37Rv genomic DNA with the primers Rv2509-U (5′-GCAGGATCCAATGCCGATACCCGCGCCC-3′) and Rv2509-D (5′-GCAGAATTCCTAGCTGCCCCCAAGCCTC-3′). The complemented strains Δ*MSMEG4722*-C and Δ*MSMEG4722*-CRv were obtained by selecting kanamycin-resistant transformants following electroporation of the mutant strain Δ*MSMEG4722* with pMV261-*MSMEG4722* or pMV261-*Rv2509* respectively. Electroporation was performed as previously described (Snapper et al., 1990).

### Lipid and Mycolic Acid Extraction and Analysis

Polar and apolar lipids were extracted from *M. smegmatis* strains and analyzed as described previously (Dobson et al., 1985). For extraction of MAMEs, both the delipidated cells and the whole-cell pellets were subjected to alkaline hydrolysis with 5% aqueous TBAH at 100°C overnight, followed by the addition of 4 ml CH_2_Cl_2_, 500 μl CH_3_I, 2 ml water, followed by mixing for 30 min. The upper aqueous phase was discarded following centrifugation, and the lower organic phase washed thrice with water and evaporated to dryness. The resulting FAMEs and MAMEs were dissolved in diethyl ether, insoluble residues were removed by centrifugation, and the ether solution evaporated to dryness and redissolved in 200 μl of CH_2_Cl_2_. Equivalent volumes of the resulting solution of FAMEs and MAMEs was subjected to TLC with silica gel plates (5735 silica gel 60F_254_; Merck, Darmstadt, Germany), developed in petroleum ether-acetone (95:5). Autoradiograms were produced by overnight exposure of Kodak X-Omat AR film to the plates to reveal [^14^C]-labeled FAMEs and MAMEs. Argentation-TLC was performed as described above after saturation of TLC plates with 10% aqueous silver nitrate solution and prior activation at 100°C for 1 hr. Lipid-Y was purified by preparative silica gel TLC with petroleum ether:ethyl acetate (98:2, v/v) and detection by spraying with ethanolic Rhodamine 6G to visualize the lipid under a 366 nm UV lamp. The area containing Lipid-Y was removed and extracted from the silica gel with diethyl ether. The extracted sample was then resolved on a second TLC plate in toluene:acetone (95:5, v/v) and purified as described above. MALDI-TOF/MS of all samples was performed with the Voyager DE-STR MALDI-TOF instrument (PerSeptive Biosystems, Framingham, MA). NMR spectra for Lipid-Y were recorded in CDCl_3_ on a Bruker DRX500 operating at 500.13 MHz for ^1^H-NMR and 125.77 MHz for ^13^C-NMR.

## Figures and Tables

**Table 1:** MALDI-TOF/MS Analysis of MAMEs Isolated from Different *M. smegmatis* Strains

	Total Carbon No. of Mycolic Acid								
	62	64	74	75	76	77	78	79	80
Untreated									

WT									
α′	952^a^	980^a^							
α			1118	1132	1146	1160^a^	1174	1188^a^	
Epoxy				1148	1162	1176^a^	1190	1204^a^	1218

NaBH_4_ treated									

WT									
α′	952^a^	980^a^							
α			1118	1132	1146	1160^a^	1174	1188^a^	
Epoxy					1162	1176^a^	1190	1204^a^	
Hydroxyl						1178		1206	1220
Δ*MSMEG4722*									
α′	952^a^	980^a^							
α			1118		1146	1160^a^		1188^a^	
α-epi^b^						1160^a^		1188^a^	
Epoxy						1176^a^	1190		
Hydroxyl						1177			
Δ*MSMEG4722*-C									
α′	952^a^	980^a^							
α			1118	1132	1146	1160^a^	1174	1188^a^	
Epoxy					1162	1176^a^	1190	1204^a^	
Hydroxyl						1178		1206	1220

The masses indicated are those of Na adducts.

a Masses of predominant species.

b α-epi is the isomer of α-mycolates.

**Table 2:** Plasmids, Bacterial Strains, and Phages Used in This Study

	Description	Reference/Source
Plasmids		

pMV261	*E. coli*-*Mycobacterium* shuttle plasmid vector with *hsp60* promoter and Kan^R^ cassette (*aph*)	Stover et al. (1991)
pMV261-*MSMEG4722*	*MSMEG4722* cloned in pMV261	This work
pMV261-*Rv2509*	*Rv2509* cloned in pMV261	This work
p0004s	Vector for cloning allelic-exchange substrates to be used for specialized transduction; contains λ phage *cos* site and Hyg^R^ cassette (*hyg*)	Gift from T. Hsu and W.R. Jacobs, Jr., Albert Einstein College of Medicine, New York
pΔ*MSMEG4722*	Derivative of p0004s designed for allelic exchange of *M. smegmatis MSMEG4722*	This work

Bacterial strains		

mc^2^155	Electroporation-proficient *ept* mutant of *M. smegmatis* strain mc^2^6	Snapper et al. (1990)
Δ*MSMEG4722*	Deletion mutant of mc^2^155 in which *MSMEG4722* is replaced by *hyg*	This work
Δ*MSMEG4722*-C	Complemented strain of Δ*MSMEG4722* containing pMV261-*MSMEG4722*	This work
Δ*MSMEG4722*-CRv	Complemented strain of Δ*MSMEG4722* containing pMV261-*Rv2509*	This work

Phages		

phAE159	Conditionally replicating shuttle phasmid derived from the lytic mycobacteriophage TM4	Bardarov et al. (2002)
phΔ*MSMEG*4722	Derivative of phAE159 obtained by cloning pΔ*MSMEG4722* into its unique PacI site	This work
